# An enhancement of the Genium™ microprocessor-controlled knee improves safety and different aspects of the perceived prosthetic experience for unilateral and bilateral users

**DOI:** 10.3389/fresc.2024.1342370

**Published:** 2024-05-02

**Authors:** Tyler D. Klenow, Russell L. Lundstrom, Arri Morris, Stan Patterson, Chad Simpson, Ernesto G. Trejo, Andreas Kannenberg

**Affiliations:** ^1^ Clinical Research & Services Department, Otto Bock HealthCare LP, Austin, TX, United States; ^2^ Clinical Services Department, Prosthetic & Orthotic Associates, Orlando, FL, United States; ^3^ Clinical Services Department, Dream Team Prosthetics, LLC, Duncan, OK, United States; ^4^ Clinical Research & Services Department, Ottobock Healthcare Products GmbH, Vienna, Austria

**Keywords:** amputee, rehabilitation, transfemoral, MPK, ADL-Q, mechatronic, biomedical

## Abstract

**Introduction:**

Bilateral microprocessor-controlled prosthetic knee (MPK) users have unique needs in traversing environmental barriers compared to unilateral users. An enhancement to the Genium™/Genium X3™ MPK which included an updated ruleset, hydraulics, and new bilateral parameter presets was made to improve safety while stumbling and the smoothness of gait for all users while also improving the experience of bilateral users. The purpose of the study was to evaluate the effectiveness of the enhancements in a sample with unilateral and bilateral amputation.

**Methods:**

A convenience sample of MPK users was recruited from two sites in the USA in two phases. Assessments included the *L*-Test of Functional Mobility, Activity-specific Balance Confidence Scale, Prosthetic Limb User Survey of Mobility, a study-specific questionnaire, and the Comparative Activities of Daily Living (ADL) Questionnaire. Statistical significance of extracted data was tested with the Wilcoxon Rank-Sum Test for independent data and Wilcoxon Signed-Rank for paired data with an *a priori* significance level of *p *< 0.05. Unilateral subjects were age-matched to the group of bilateral subjects for between-groups and within-groups analyses.

**Results:**

Twenty-six subjects (*n *= 26) were enrolled. Stumble frequency reduced 85% from 16.0 ± 39.7 to 2.4 ± 2.3 (*p *= 0.008) between baseline and final assessment overall. The bilateral group reported 50% (*p* = 0.009) and 57% (*p* = 0.009) greater relative improvement in patient-reported ease and safety, respectively, of completing ADLs compared to the unilateral group. The unilateral group reported residual limb pain and low back pain reduced from 2.3 to 1.4 (*p* = 0.020) and 3.8 to 1.8 (*p* = 0.027), respectively, whereas the bilateral group did not.

**Discussion:**

Substantial reductions in stumbles, residual limb pain, and back pain were shown overall. These reductions were driven by the unilateral group who also showed improvements in comfort, exertion, and concentration while walking. The enhancements to the knee likely reduced some gait asymmetry for unilateral users. Improvements in patient-reported ease and safety of completing ADLs were shown overall and were driven by the bilateral group. This study shows further improvement in patient experience is achievable through innovation in MPK technology even for patients who appear to be functioning well.

## 1 Introduction

**Figure 1:**
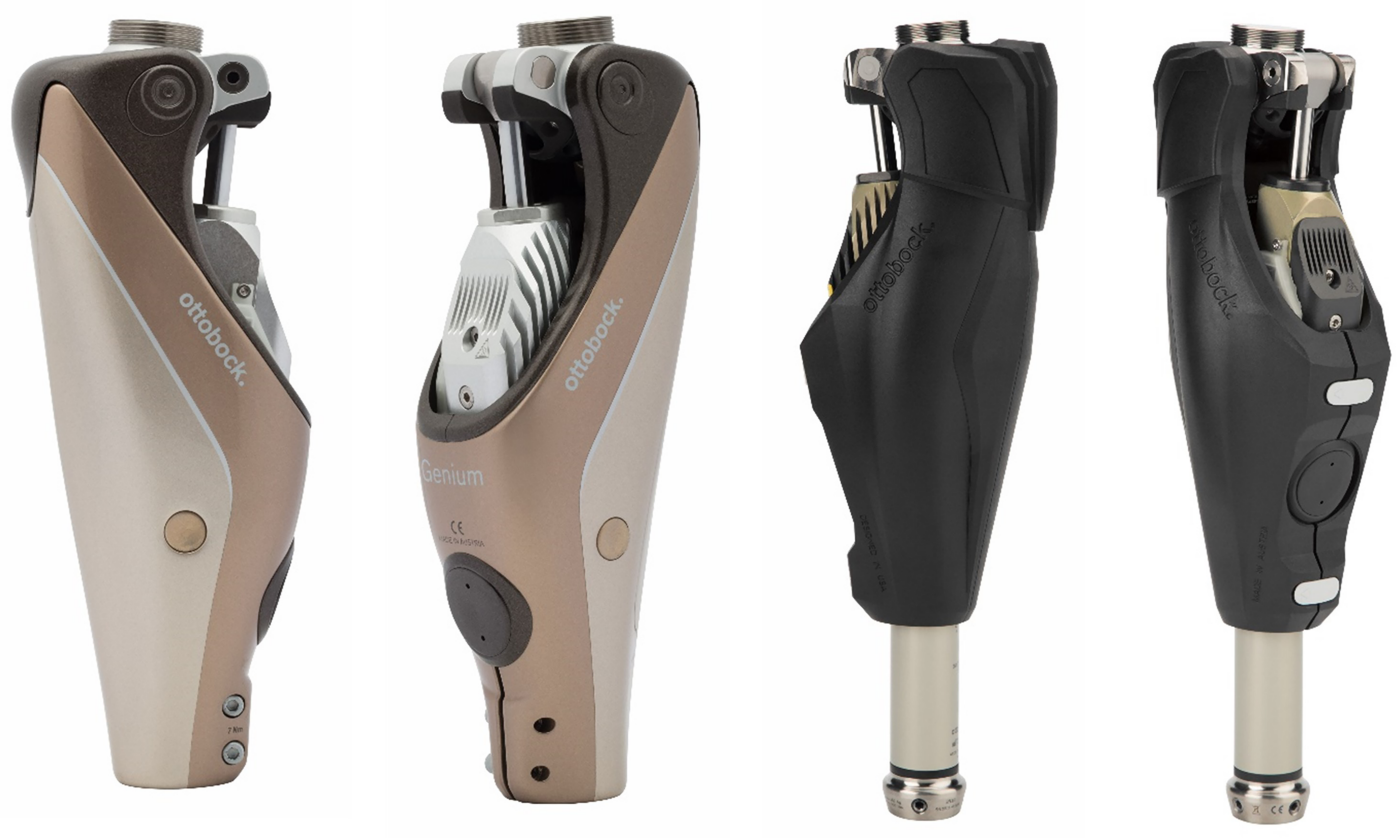
Genium and Genium X3 images, reprinted with permission from Otto Bock Healthcare LP.

Individuals with lower extremity amputation (LEA) are a relatively small population in allied health. Those with transfemoral amputation (TFA) constitute a smaller proportion of the population compared to patients with transtibial amputation (TTA), and those with bilateral TFA make up an even smaller proportion yet (1). TFA patients often have poor rehabilitation outcomes due to the absence of two major biological joints in both lower extremities (2, 3). Significant efforts have brought technological advancements to patients with TFA in the form of microprocessor-controlled knee (MPK) joints (4). Most MPKs on the market today utilize some application of a hydraulic cylinder which dampens flexion and extension of the joint during stance and swing phases of gait and standing (5). The degree of dampening is controlled by a microprocessor which accepts input from various sensors and opens or closes hydraulic valves in response to a decision tree called a ruleset. The Genium™ (Ottobock Healthcare Products GmbH, Vienna, AT) (Figure 1) was introduced in 2011 containing an advanced control concept, additional sensors, and improved algorithms enabling a range of new functions for MPK users (4). Specific technological modifications for bilateral users have not yet been developed, however.

Invariably, the needs of bilateral and unilateral users differ in response to similar gait events due to absence or presence of a sound limb. The most notable gait events are ramp and stair negotiation where the unilateral prosthesis users can use the sound limb to control the speed of slope or stair descent and rely on it as a primary stability point (6). However, the bilateral user is solely reliant on the capability of assistive technology to complete these activities of daily living (ADLs) (5). Most commercially available MPKs have a programming selection for bilateral users, but this option often alters only the appearance of the graphical user interface (GUI) for the device, the reporting function, or the ability to connect the GUI to multiple devices but not the ruleset parameters. A functional difference between groups is thereby created because the identical functionality for both groups results in a relatively poorer prosthetic experience for patients with bilateral TFA than with unilateral TFA (3). The lack of specific functional options may force bilateral users to preemptively avoid problematic situations (7). Situation avoidance leads to activity avoidance and reductions in social and community participation which ultimately results in reduced quality of life (7–10). However, these reductions may be avoided if features are created to improve the experience of bilateral users because previous research has shown relative fulfilment of rehabilitative potential has a greater impact on mental health and quality of life than the laterality of amputation (11, 12).

A recent enhancement to the Genium and Genium X3 was made to improve safety during stumbling and improve everyday walking for users. An additional objective was to introduce bilateral parameter presets for the rulesets of these MPKs. Therefore, the purpose of this study was to investigate the effectiveness of the enhancements to improve safety during stumbling and to improve everyday walking in a group of subjects with both unilateral and bilateral TFA. It was hypothesized that the enhancements would reduce stumbles and falls and would improve gait stability and comfort overall. An additional purpose was to investigate whether the introduction of bilateral parameter presets improve the prosthetic experience of bilateral prosthesis users. It was hypothesized that the enhancements would result in greater improvements in patient-reported ease and safety of ADL completion in a group of bilateral users compared to a control group of unilateral users following the final update.

## 2 Materials and methods

**Figure 2:**
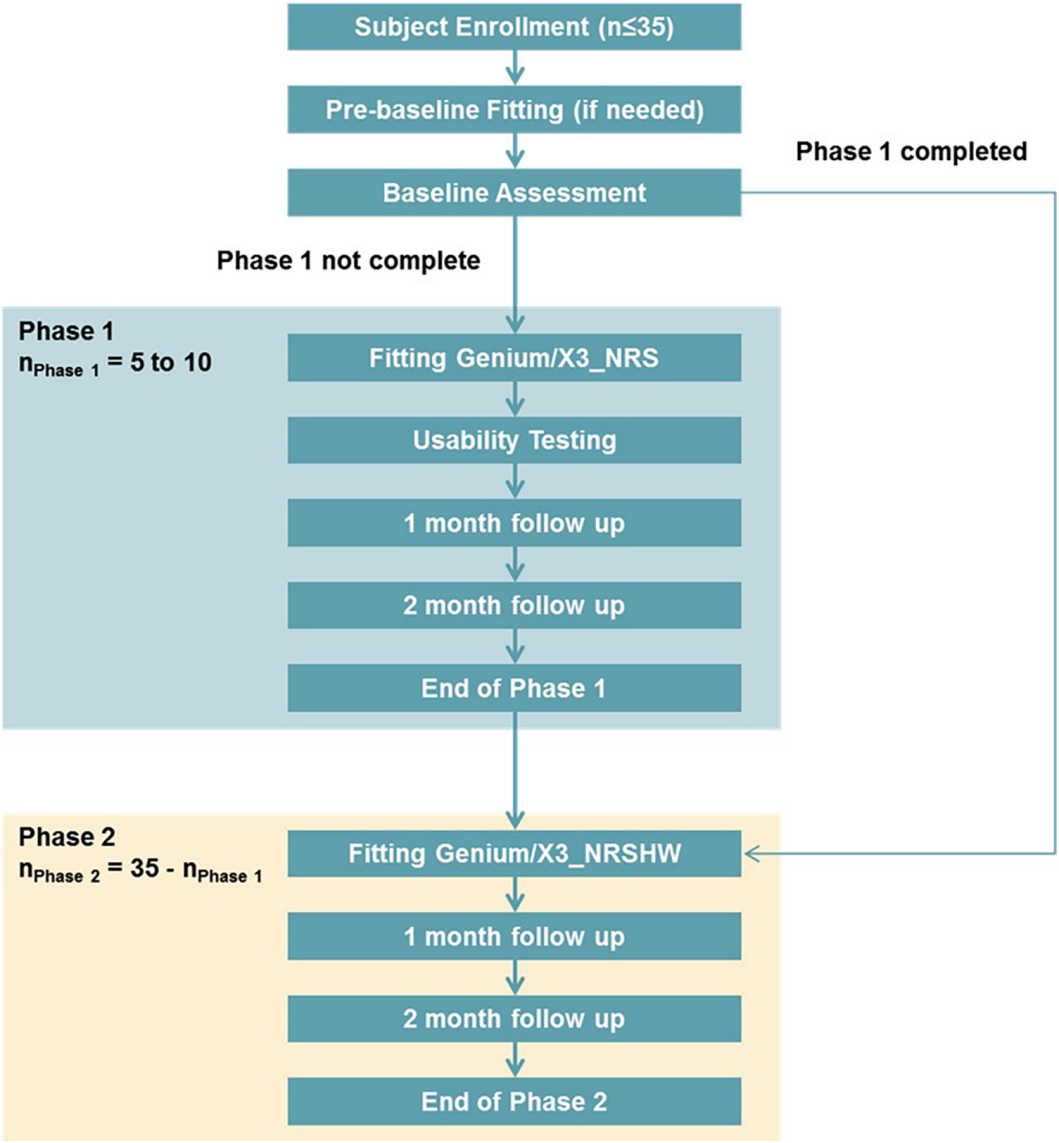
Study subject flow.

The Institutional Review Board approval of the study protocol was provided by WCG IRB (WCG #20171027). The clinical trial was divided into two phases (Figure 2). Phase I included proof of concept testing of developmental ruleset changes in a small group of MPK end-users. Investigational Genium and Genium X3 knees featured enhanced rulesets including specific parameter presets for bilateral users in Phase I. Phase II included a larger sample for testing prior to commercialization. Devices in phase II featured the specific parameter presets for bilateral subjects as well as updated rulesets and hydraulics for all users. Direct feedback was provided by subjects and prosthetists to design engineers at the beginning of phase II. Indirect feedback was provided through outcome measures (OMs) collected at various points as detailed below. This feedback was integrated prior to implementation of the final ruleset update which was uploaded to all investigational MPKs two months prior to the final assessment.

### 2.1 Subject recruitment

A convenience sample of MPK users was recruited from two participating clinics in Oklahoma and Florida in the United States. Inclusion criteria were:

• History of TFA, knee disarticulation (KD), or hip disarticulation (HD)
• 6 months prior experience with a Genium or Genium X3
• Current prosthetic use >8 h per day
• Demonstrated ability to walk at different speeds
• Ability to ascend and descend ramps and stairs
• Medicare Functional Classification Level K2, K3 or K4
• Use of a compatible conventional prosthetic interface (socket)
• Willingness to use the study MPK with a smartphone app

Exclusion criteria were:

• <18 years old
• Serious health risks which may prevent participation (e.g., unstable cardiovascular conditions, terminal cancer, etc.)
• History of chronic skin breakdown of residual limb
• Falls once per week for reasons not related to prosthetic use (e.g., vestibular disorders)
• Current pregnancy
• Current or anticipated participation in another clinical trial

Sites were asked to enroll a minimum of three subjects with bilateral TFA or KD in Phase I and at least seven bilateral subjects in Phase II for a total of 10. Sites were asked to enroll seven subjects with unilateral amputation in Phase I and at least 7 in phase II for a total of 14. Subjects from Phase I continued participation through Phase II.

### 2.2 Device assignment, fitting, and assessments

Following informed consent, each subject's existing prosthesis was evaluated for fit and function by the site principal investigator and the sponsor's clinical specialist. Subjects then completed a screening assessment to ensure compliance with inclusion and absence of exclusion criteria. All existing prostheses had a commercially-available Genium or Genium X3 knee and a prosthetic foot from the Triton™ product line (Ottobock SE & Co. KGaA; Duderstadt, GER) or the TaiLor Made™ (Prosthetic & Orthotic Associates; Orlando, FL, USA) except for one subject who used a Flex Foot Junior (Ossur, Reykjavik, ISL) due to foot size limitations. The baseline assessment included collection of demographics, a general health questionnaire, several validated outcome measures (OMs), and the Study-Specific Questionnaire (SSQ) described below in Section 2.3.

Following the baseline assessment, subjects entered Phase I where they were provided an investigational Genium or Genium X3, corresponding to their existing knee model, with the enhanced ruleset. Bilateral subjects used the bilateral parameter presets as part of their initial programming. In Phase II, subjects were fit with a new investigational Genium or Genium X3 with an enhanced ruleset and updated hydraulics. Bilateral subjects again used the bilateral parameter presets as part of their initial programming. At the end of Phase II, subjects completed the protocol with the assessment of the same outcome measures completed at baseline along with the comparative Activities of Daily Living Questionnaire (ADL-Q) described below in Section 2.3.

### 2.3 Outcome measures

The OMs used in the baseline and final assessments are described below:

Subject-reported stumbles and falls were collected by asking subjects to recall the frequencies of each in the previous 8 weeks using the following categories: never, once, 2–5 times, once per week, 2–5 times weekly, once per day, or 2–5 times per day. The answers were converted to estimated numbers of falls or stumbles over the previous 8-week period, taking the midpoint of the range of each response category where applicable.

The Numeric Pain Rating Scale (NPRS) is a single-point evaluation of the highest pain experienced in the last week at the low back and in the residual limb on a continuous scale from 0 to 10. It has a minimally clinically important difference (MCID) of 1 for individuals with chronic pain and other musculoskeletal disorders (13–17). Excellent internal consistency has been demonstrated for young (Cronbach alpha = 0.88) and elderly subjects (Cronbach alpha = 0.87) alike as well as excellent inter-rater reliability (18).

The *L*-Test is a modified version of the Timed-up-and-go (TUG) test that increases the total distance and number of turns. Experienced MPK users were expected to encounter a ceiling effect or insufficient challenge in the TUG test. Since the *L*-Test has reduced the ceiling effect of the TUG by 52% and also highly correlates with it (Pearson *r* = 0.93), the *L*-test was considered more appropriate for the subjects in this study (19). The *L*-test has an MCID of 4.5 s for individuals with lower extremity amputation as established by Rushton et al. (20) and a fall-risk threshold of >25.5 s for healthy elderly people (21).

The Activities-specific Balance Confidence (ABC) Scale is 16-item self-reported measure designed to identify balance confidence issues (22). Each of the 16 activities is rated on a 10-point scale between 0% and 100% in 10% increments, with greater scores indicating better balance confidence. The total score is then averaged across the 16 activities. A fall-risk threshold of <67% has been established for elderly people (23).

The Prosthetic Limb Users Survey of Mobility (PLUS-M) is a self-report instrument for measuring mobility of adults with lower limb amputation. The PLUS-M 12-item short form provides T-scores that range from 21.8 to 71.4 (24). Higher T-scores indicate better mobility. A T-score of 50 is equal to the mean of the development sample and every 10 points correspond approximately to one standard deviation (25). For example, a patient with a PLUS-M T-score of 60 is one standard deviation above the average respondent from the original (*n* = 1,091) sample.

**Figure 3:**
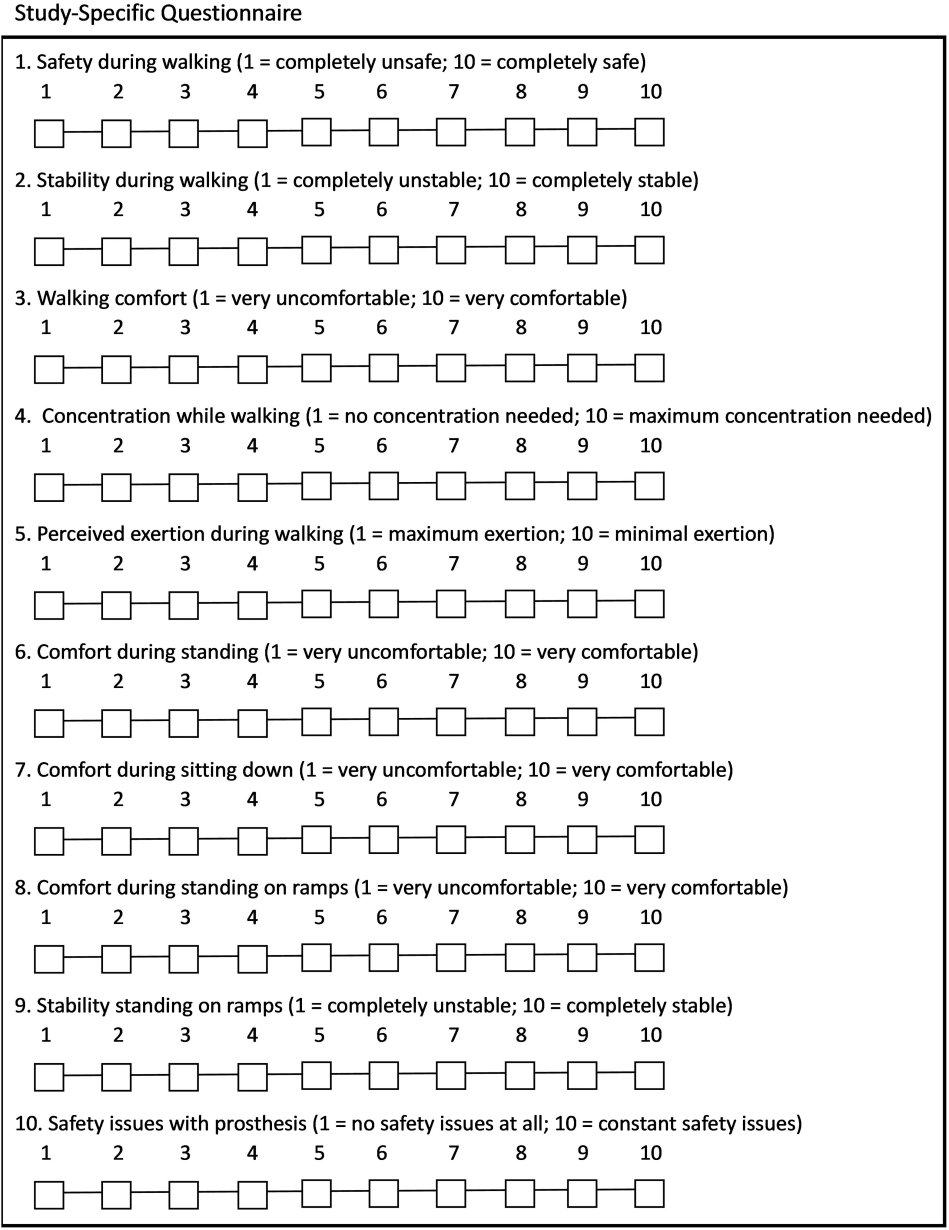
Study-specific questionnaire.

A study-specific questionnaire (SSQ) was created and used to evaluate the effect of specific aspects of the enhancements on subjects' experience during common prosthetic tasks. Questions in the SSQ included patient-reported ratings of walking safety, walking stability, walking comfort, concentration while walking (autowalk), exertion while walking, standing comfort, sitting comfort, comfort standing on ramps, stability standing on ramps, and use of the stair and ramp functions rated on a 10-point scale (Figure 3). The SSQ was administered at baseline and final assessments as well as at various points throughout the study period to provide feedback to product developers. Only baseline and final scores will be reported in this article.

The Comparative Activities of Daily Living Questionnaire (ADL-Q) is a 45-item questionnaire related to ADLs grouped into seven categories: personal care and dressing, family and social roles, leisure time activities, mobility, transportation, health related exercise, and other activities (26). Subjects reported perceived ease and safety of completing ADLs on a 5-point Likert scale ranging from much improvement with the existing knee joint (−2 pts) to much improvement with investigational knee joint (+2 pts). The ADL-Q has been used in studies evaluating advanced MPKs in the past (4, 26). A threshold for clinically significant change of 0.5 was suggested by Kannenberg et al. in 2013 (26). The comparative ADL-Q was administered at the final assessment.

Subjects were also asked if they used functions of the knee including yielding down slopes and stair ascent mode both for ascending stairs and stepping over obstacles. These questions were asked at the baseline and final assessments.

### 2.4 Statistical analysis

All results were described with measures of central tendency (e.g., means, standard deviations). Comparisons were made between the baseline and final assessments on aggregate. A subset of unilateral subjects was age-matched to the group of bilateral subjects for a between-groups analysis. Means and standard deviations at baseline and final assessment between groups and within each of the age-matched groups were calculated and compared.

Statistical significance was tested with the Wilcoxon Rank-Sum Test for independent data and Wilcoxon Signed-Rank test for paired data with an *a priori* significance level of *p *< 0.05. Calculations were completed in Python statistical analysis software SciPy 3.11 (Python Software Foundation; Fredericksburg, VA, USA).

## 3 Results

Twenty-six (*n* = 26) subjects were enrolled in the study. Ten (*n* = 10) were enrolled in Phase I and an additional 16 were enrolled in Phase II. One unilateral subject dropped out due to worsening of pre-existing back pain in Phase II and was not included in the analysis. One subject had a history of unilateral TFA and contralateral TTA and was excluded from the between-group analysis but included in the aggregate analysis. Demographic data is shown in Table 1.

**Table 1:** Demographics.

Demographic	Aggregate	Unilateral (age-matched)	Bilateral
Number of subjects	26	9	9
Gender	2 Female, 24 Male	1 Female, 8 Male	0 Female, 9 Male
Age (years)	35.1 ± 12.6	29.3 ± 7.1	29.3 ± 5.1
Prosthetic experience			
Mean time since amputation (years)	15.0 ± 12.2	11.4 ± 5.6	12.3 ± 11.3
Mean time using MPK (years)	3.7 ± 2.2	4.4 ± 2.8	2.8 ± 1.9
Etiology			
Trauma	20	8	6
Congenital	3	1	2
Tumor	1		
Vascular	1		
Rhabdomyolysis	1		1
Amputation level			
Hip disarticulation	1		
Transfemoral	22	8	7
Knee disarticulation	3	1	2
Study knee			
Genium	8	3	4
X3	18	6	5

### 3.1 Aggregate analysis

**Figure 4:**
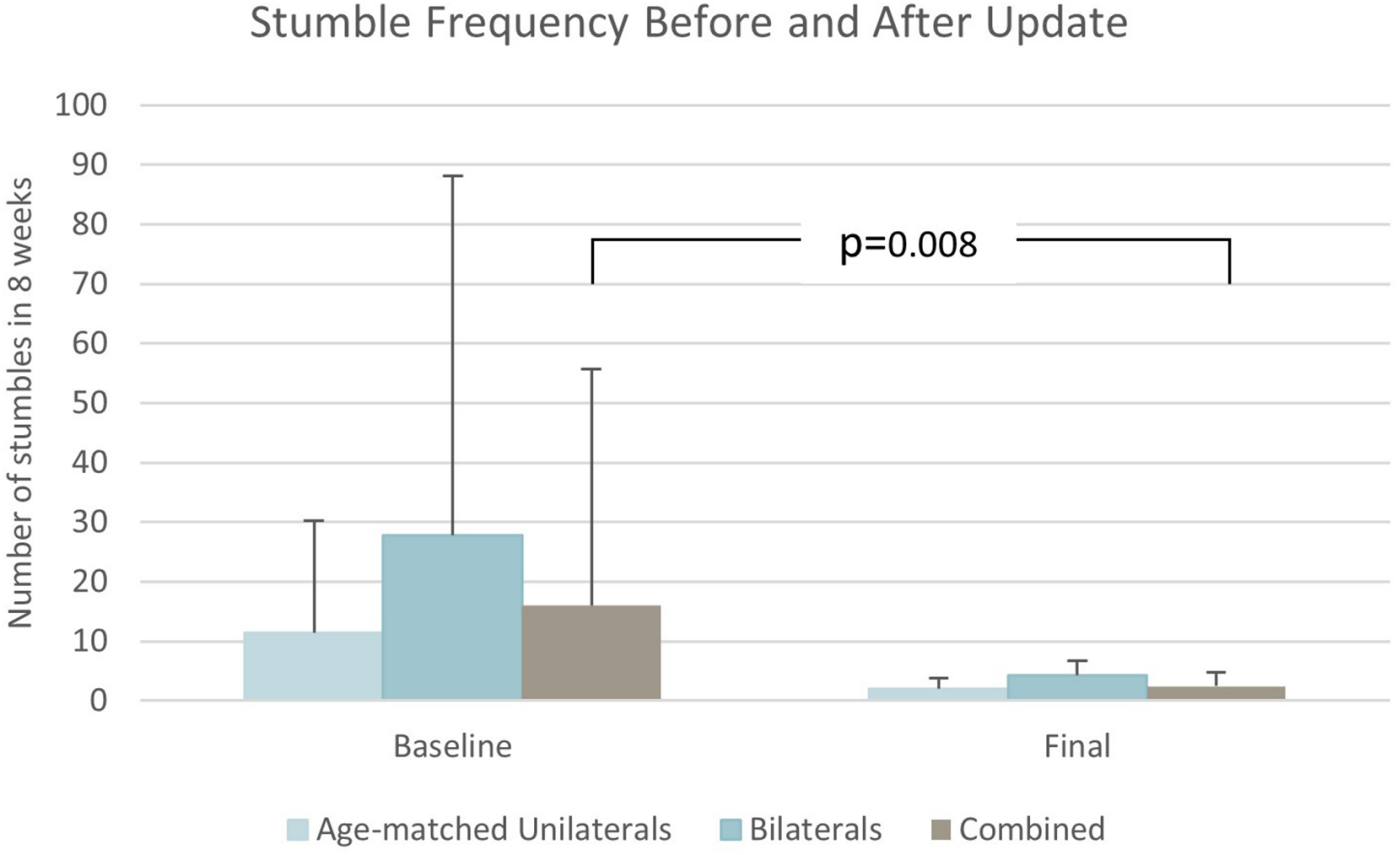
Stumble frequency results.

**Figure 5:**
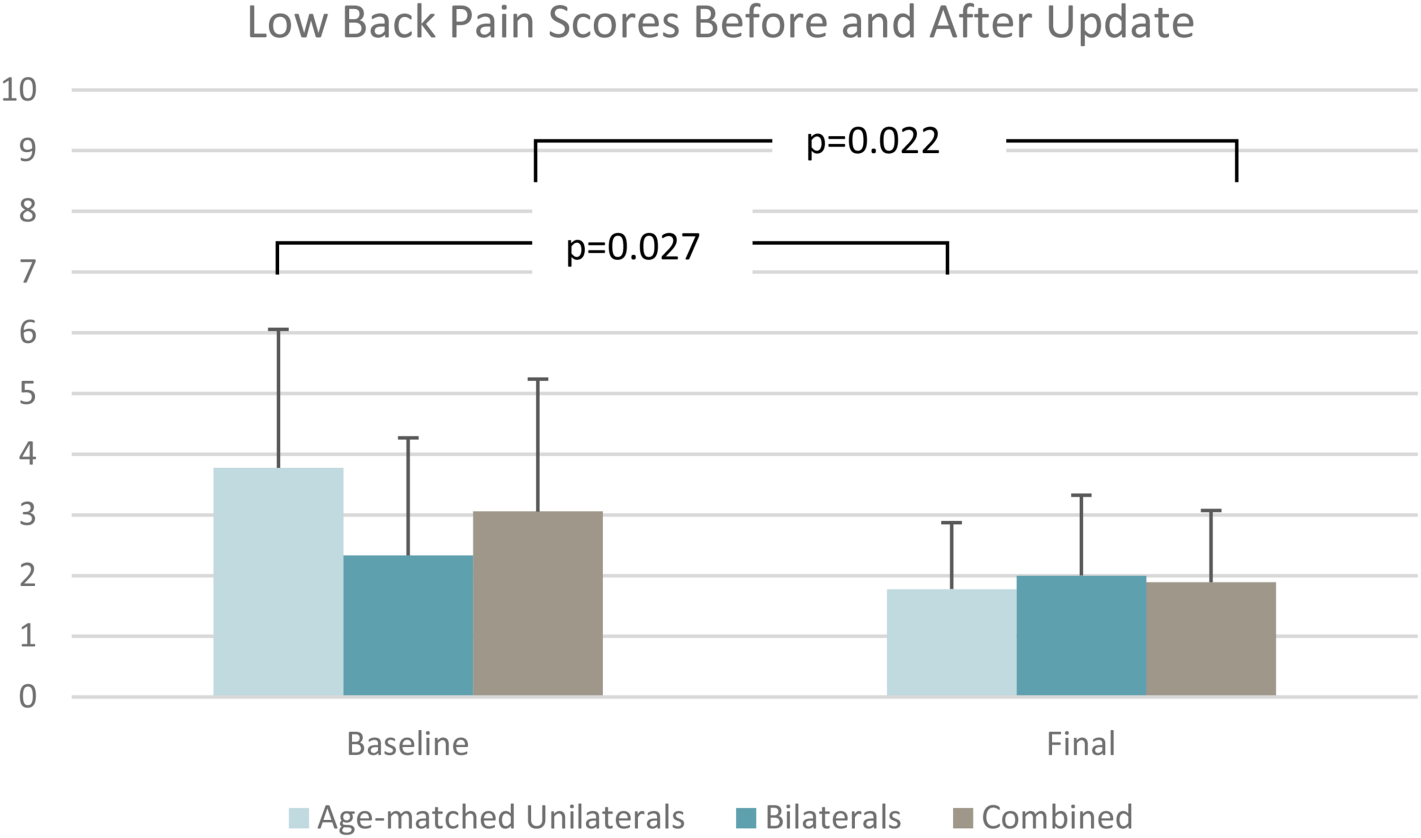
Low back pain scores.

**Figure 6:**
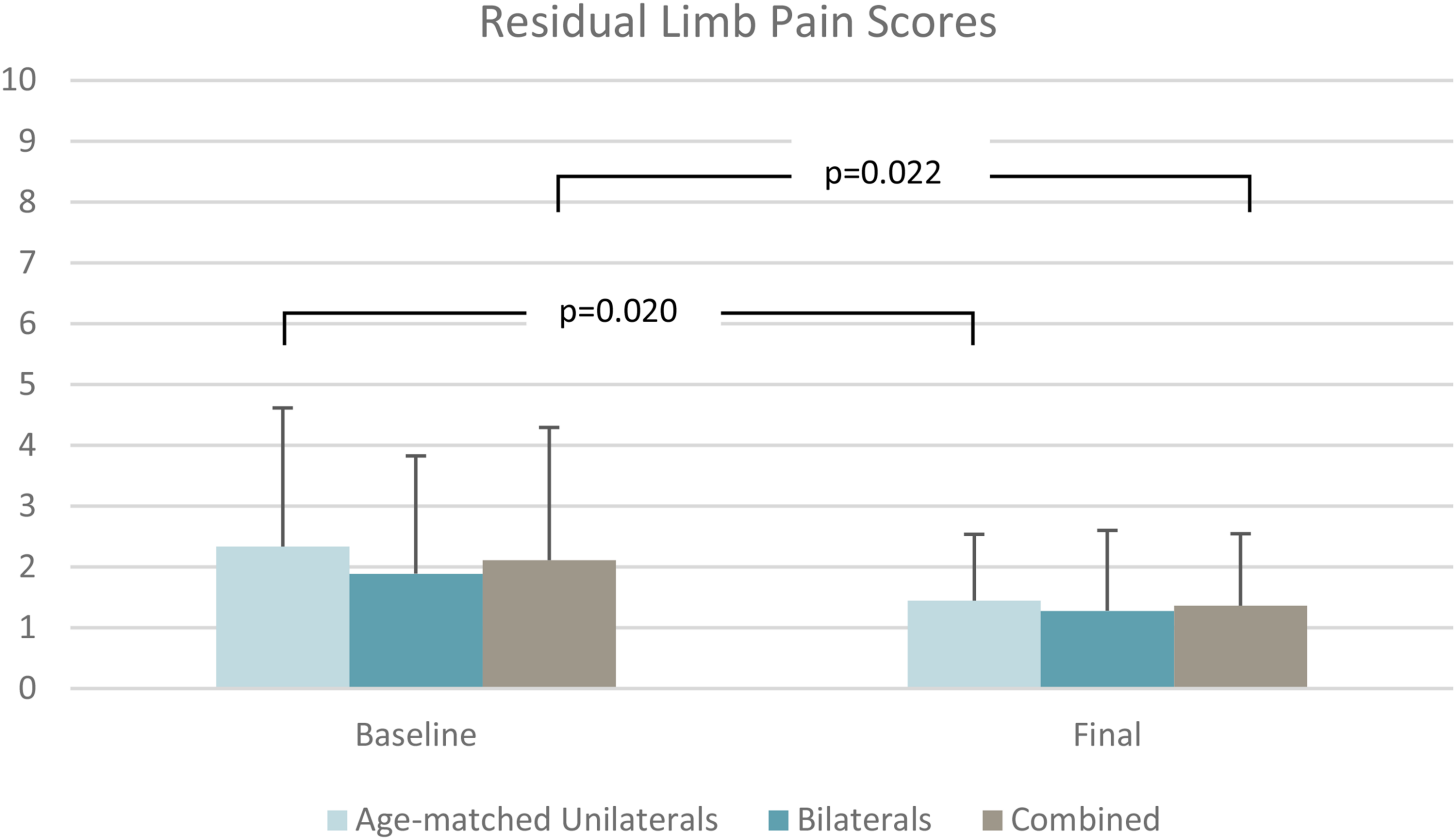
Residual limb pain scores.

Stumble frequency (Figure 4) reduced significantly by 85% from baseline to final assessments (*p* = 0.008) as shown in Table 2. Fall frequency was low at baseline already, so the observed further reduction at final assessment did not attain statistical significance (Table 2). Low back pain (*p* = 0.022) (Figure 5) and residual limb pain (*p* = 0.002) (Figure 6) were reduced as shown in Table 3. No statistically significant change was observed in *L*-Test, PLUS-M or ABC (Table 3). In the SSQ, subjects reported statistically significant improvements ranging from +0.7 to +1.6 for walking safety (*p* = 0.046), walking comfort (*p* = 0.002), exertion while walking (*p* = 0.010), concentration while walking (*p* = 0.006), standing comfort (*p* = 0.010), sitting comfort (*p* = 0.040), stability standing on ramps (*p* = 0.001), and overall prosthetic safety (*p* = 0.009) (Table 4). In the comparative ADL-Q (Figure 6), clinically meaningful improvements (>0.5) were demonstrated in both safety and ease of ADL completion in the areas of Family Role, Social and Leisure Activities, Shopping, Mobility, Transportation, Health-Related Exercise, and Other Activities as shown in Table 5.

**Table 2:** Falls and Stumbles in the previous 8 weeks.

Measure	Aggregate		Age-matched Unilateral		Bilateral	
	Baseline	Final	Baseline	Final	Baseline	Final
Falls	1.16 ± 1.41	0.74 ± 1.70	0.72 ± 1.15	0.22 ± 0.44	1.89 ± 1.51	1.72 ± 2.59
	Δ = −0.42; *p* = 0.133		Δ = −0.50; *p* = 0.179		Δ = −0.17; *p* = 0.713	
Stumbles	16.0 ± 39.7	2.4 ± 2.3	11.3 ± 18.9	1.9 ± 1.8	27.8 ± 60.3	4.2 ± 2.4
	Δ = −13.5; *p* = 0.008		Δ = −9.4; *p* = 0.115		Δ = −23.6; *p* = 0.246	

Δ = change from Baseline after update.

**Table 3:** Results for all subjects, age-matched unilateral group, and bilateral subjects.

Measure	Aggregate		Age-matched unilateral		Bilateral	
	Baseline	Final	Baseline	Final	Baseline	Final
*L*-test (sec)	24.0 ± 4.7	23.6 ± 4.8	21.0 ± 4.7	20.9 ± 4.6	26.1 ± 4.7***	25.7 ± 4.7*
	Δ = −0.5; *p *= 0.462		Δ = −0.1; *p *= 0.932		Δ = −0.4; *p *= 0.750	
ABC (%)	86.6 ± 13.5	88.8 ± 10.9	87.8 ± 13.8	92.2 ± 6.2	86.5 ± 17.6	85.3 ± 14.4
	Δ = + 2.2; *p *= 0.201		Δ = + 4.4; *p *= 0.164		Δ = −1.2; *p *= 0.400	
PLUS-M (t-score)	57.4 ± 7.7	57.2 ± 6.1	57.4 ± 7.6	59.6 ± 6.6	59.1 ± 8.3	55.6 ± 6.2
	Δ = −0.2; *p *= 0.783		Δ = + 2.2; *p *= 0.426		Δ = −3.4; *p *= 0.249	
Residual limb pain	2.1 ± 1.4	1.5 ± 1.0	2.3 ± 1.2	1.4 ± 0.5	1.9 ± 1.7	1.3 ± 0.8
	Δ = −0.6; *p *= 0.002		Δ = −0.9; *p *= 0.020		Δ = −0.6; *p *= 0.109	
Low back pain	2.9 ± 2.1	2.0 ± 1.3	3.8 ± 2.3	1.8 ± 1.1	2.3 ± 1.9	2.0 ± 1.3
	Δ = −1.0; *p *= 0.022		Δ = −2.0; *p *= 0.027		Δ = −0.3; *p *= 0.593	

Δ = change from Baseline after update.

^*^p < 0.05, ^**^p < 0.01, ^***^p < 0.001, for between group comparisons (age-matched unilateral vs. bilateral).

**Table 4:** Results for SSQ items.

Item	Aggregate		Age-matched unilateral		Bilateral	
	Baseline	Final	Baseline	Final	Baseline	Final
Exertion during walking	3.0 ± 2.2	1.8 ± 1.1	3.2 ± 2.0	1.7 ± 0.7	2.8 ± 2.2	2.1 ± 1.6
	Δ = −1.2; *p* = 0.012		Δ = −1.6; *p* = 0.041		Δ = −0.7; *p* = 0.380	
Concentration during walking	2.4 ± 1.8	1.7 ± 1.4	2.2 ± 1.3	1.2 ± 0.7	2.3 ± 2.3	2.3 ± 2.2*
	Δ = −0.8; *p* = 0.006		Δ = −1.0; *p* = 0.024		Δ = 0.0; *p* = 1.000	
Walking safety	8.4 ± 2.6	9.7 ± 0.6	9.6 ± 0.7	9.7 ± 0.4	8.0 ± 3.0	9.8 ± 0.4
	Δ = + 1.2; *p* = 0.046		Δ = + 0.1; *p* = 0.705		Δ = + 1.8; *p* = 0.141	
Walking stability	8.7 ± 2.1	9.6 ± 0.6	9.3 ± 0.7	9.7 ± 0.5	7.7 ± 3.2	9.6 ± 0.7
	Δ = + 0.9; *p* = 0.060		Δ = + 0.3; *p* = 0.257		Δ = + 1.9; *p* = 0.136	
Walking comfort	8.3 ± 2.1	9.6 ± 0.6	8.6 ± 0.9	9.7 ± 0.4	7.8 ± 3.2	9.7 ± 0.7
	Δ = +1.4; *p* = 0.002		Δ = + 1.1; *p* = 0.020		Δ = + 1.9; *p* = 0.136	
Standing comfort	8.5 ± 1.8	9.5 ± 0.8	8.7 ± 1.3	9.4 ± 0.9	8.2 ± 2.7	9.6 ± 0.7
	Δ = + 1.0; *p* = 0.015		Δ = + 0.8; *p* = 0.068		Δ = ±1.3; *p* = 0.197	
Sitting comfort	8.7 ± 2.3	9.4 ± 1.1	9.2 ± 1.1	9.3 ± 0.5	8.8 ± 3.0	9.8 ± 0.4
	Δ = + 0.7; *p* = 0.039		Δ = + 0.1; *p* = 0.564		Δ = + 1.0; *p* = 0.414	
Stability standing on ramps	7.0 ± 2.1	8.6 ± 1.7	8.1 ± 1.5	9.4 ± 0.8	5.8 ± 2.7	7.7 ± 2.5
	Δ = + 1.6; *p* = 0.001		Δ = + 1.3; *p* = 0.031		Δ = + 1.9; *p* = 0.074	
Comfort standing on ramps	7.8 ± 1.8	8.4 ± 1.8	8.2 ± 1.6	9.0 ± 0.9	7.3 ± 2.3	7.7 ± 2.5
	Δ = + 0.6; *p* = 0.232		Δ = + 0.8; *p* = 0.161		Δ = + 0.3; *p* = 1.000	
Overall prosthesis safely	8.2 ± 2.8	9.8 ± 0.4	8.6 ± 2.6	9.7 ± 0.4	8.0 ± 3.3	10.0 ± 0.0
	Δ = + 1.6; *p* = 0.009		Δ = + 1.1; *p* = 0.236		Δ = + 2.0; *p* = 0.109	

Δ = change from Baseline after update.

* *p* < 0.05, for between group comparisons (age-matched unilateral vs. bilateral).

**Table 5:** ADL-Q results.

Category	Ease			Safety		
	Aggregate	Age-matched unilateral	Bilateral	Aggregate	Age-matched unilateral	Bilateral
Personal care and dressing	0.09 ± 0.35	0.06 ± 0.33	0.14 ± 0.42	0.1 ± 0.36	0.06 ± 0.23	0.17 ± 0.51
Family role	0.99 ± 0.86	0.74 ± 0.81	1.56 ± 0.70***	1.08 ± 0.81	0.81 ± 0.62	1.56 ± 0.75***
Social and leisure activities	1.07 ± 0.74	0.89 ± 0.65	1.51 ± 0.73***	1.1 ± 0.78	0.89 ± 0.75	1.58 ± 0.69***
Shopping	1.07 ± 0.74	0.89 ± 0.65	1.51 ± 0.73***	1.1 ± 0.78	0.89 ± 0.75	1.58 ± 0.69***
Mobility	0.97 ± 0.78	0.88 ± 0.74	1.25 ± 0.81***	1.02 ± 0.81	0.9 ± 0.76	1.32 ± 0.82***
Transportation	0.6 ± 0.76	0.49 ± 0.69	0.93 ± 0.89*	0.63 ± 0.79	0.51 ± 0.69	1.00 ± 0.93*
Health related exercise	0.8 ± 0.82	0.73 ± 0.69	1.05 ± 0.85	0.8 ± 0.84	0.57 ± 0.77	1.22 ± 0.92**
Other activities	0.7 ± 0.76	0.65 ± 0.70	0.91 ± 0.82	0.71 ± 0.77	0.62 ± 0.73	0.98 ± 0.89*

* *p* < 0.05.

** *p* < 0.01.

*** *p* < 0.001, for between group comparisons (age-matched unilateral vs. bilateral).

### 3.2 Between-groups analysis

**Figure 7:**
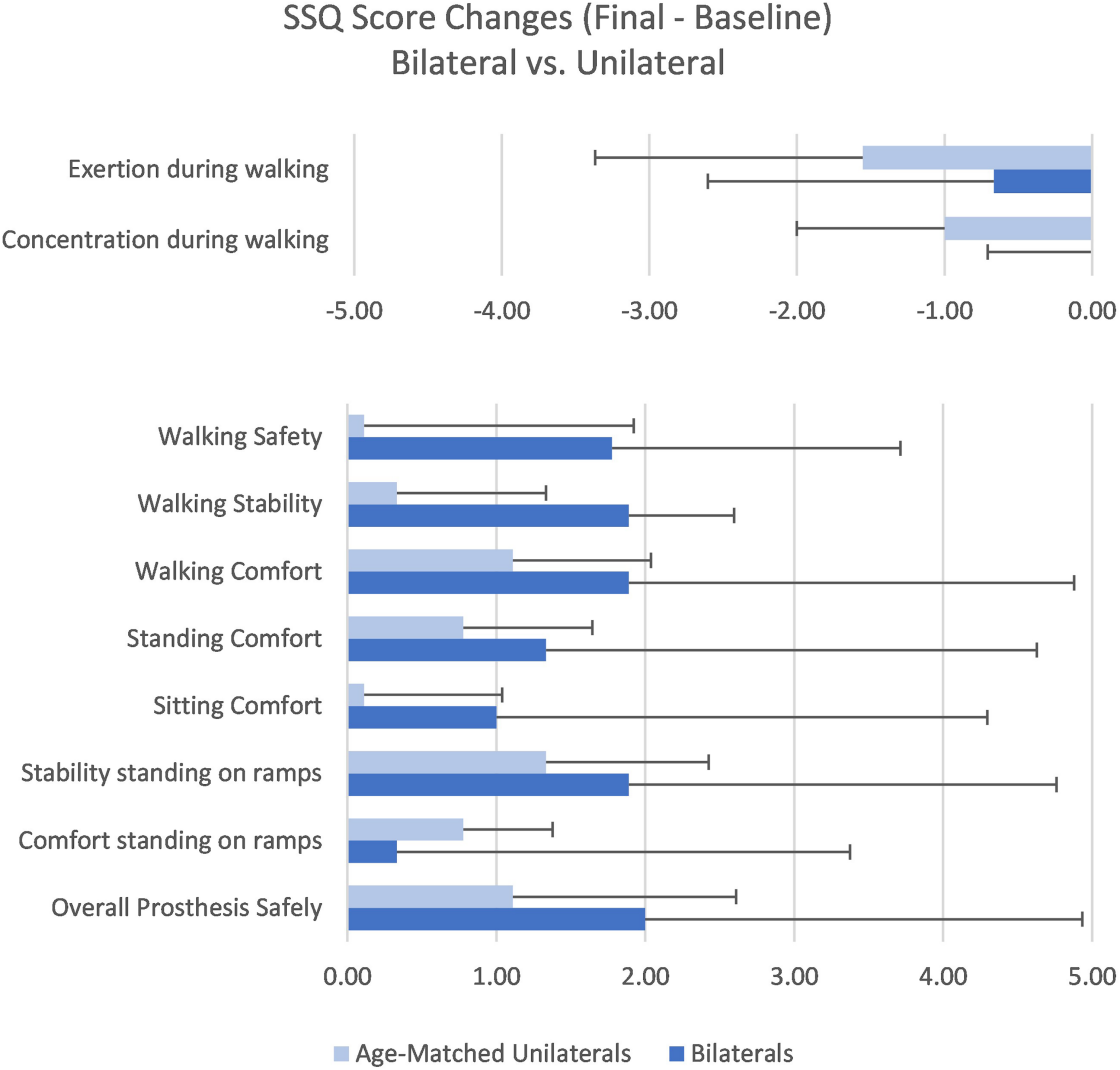
Study-specific questionnaire change scores.

**Figure 8:**
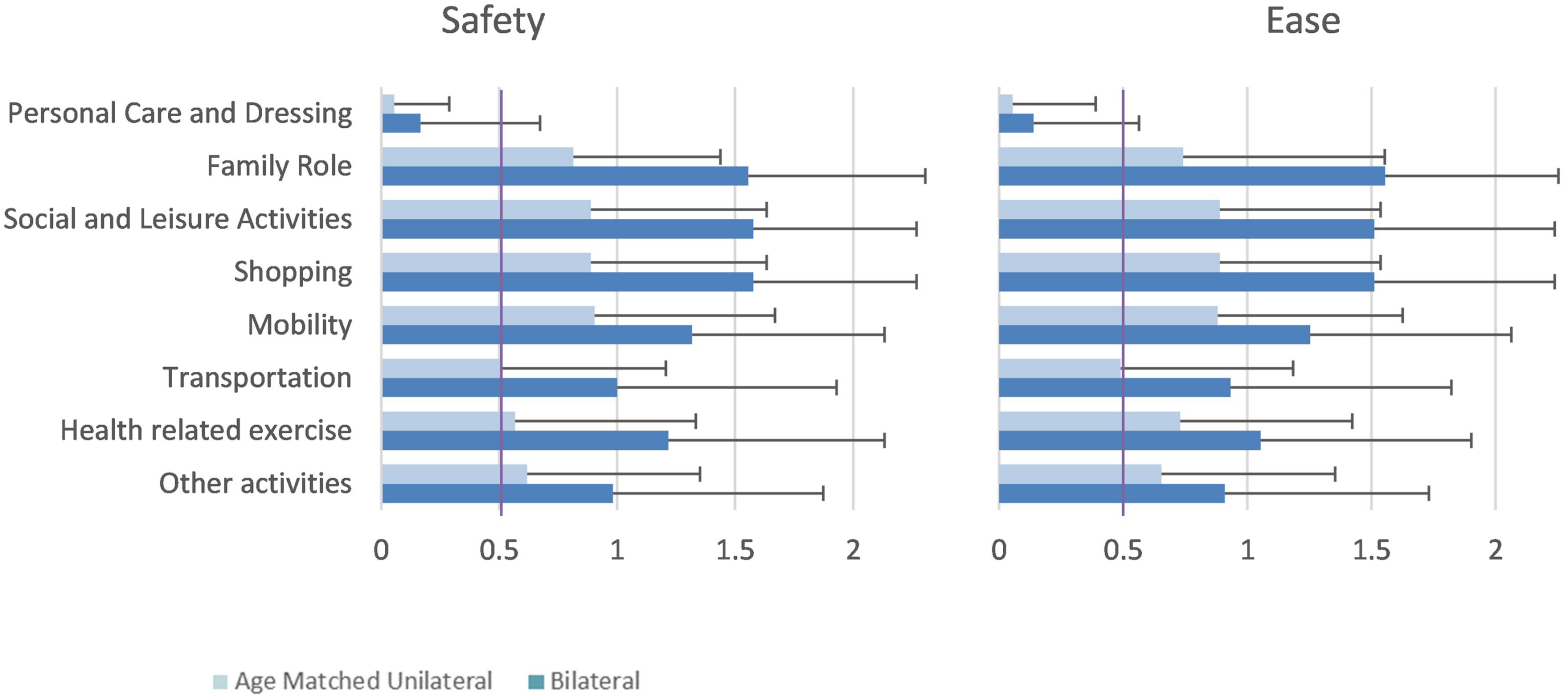
Comparative ADL-Q results.

No significant differences between unilateral and bilateral users were observed for stumble or fall frequency between groups (Table 2). Subjects with unilateral amputation demonstrated faster times on the *L*-Test than the bilateral group at baseline (*p* = 0.007) and final assessments (*p* = 0.027) as shown in Table 3. No statistically significant differences were shown between groups for the PLUS-M or ABC. For the SSQ, the unilateral group reported lower scores (Figure 7) than the bilateral group only on the item of perceived concentration while walking at the final assessment (*p* = 0.026) as shown in Table 4; no other statistically significant differences were shown. Regarding the comparative ADL-Q, the bilateral group reported 50% greater improvement in ease (*p* = 0.009) and 57% greater improvement in safety (*p* = 0.009) of ADL execution compared to the unilateral group with the investigational (Table 5). In the activity categories (Figure 8), the bilateral group improved more than the unilateral group in ease and safety with statistically significant relative improvements in ease of Family Role (*p* = 0.0006), Social and Leisure Activities (*p* = 0.0001), Shopping (*p* = 0.0001), Mobility (*p* = 0.0004), and Transportation (*p* = 0.027), as well as safety in Family Role (*p* = 0.0005), Social and Leisure Activities (*p* = 0.00008), Shopping (*p* = 0.00008), Mobility (*p* = 0.0001), Transportation (*p* = 0.021), Health-Related Exercise (*p* = 0.004), and Other Activities (*p* = 0.043). Bilateral subjects reported greater utilization of optimized stair ascent and stepping over obstacles compared to the age-matched unilateral group as shown in Table 6.

**Table 6:** Utilization of MPK functions.

Function	Unilateral			Bilateral		
	Baseline	Final	±	Baseline	Final	±
Knee yielding down slopes	9	8	−1	9	9	0
Optimized stair ascent						
- Stair ascent	2	3	+1	7	8	+1
- Stepping over obstacles	3	5	+2	6	6	0

### 3.3 Within-groups analysis

Subjects with unilateral TFA experienced reduced low back pain (*p* = 0.027) (Figure 5) and residual limb pain (*p* = 0.020) (Figure 6) from baseline to final assessment whereas the bilateral group did not (Table 3). Regarding the SSQ, subjects with unilateral TFA reported improved walking comfort (*p* = 0.020), exertion while walking (*p* = 0.040), concentration while walking (*p* = 0.023), and stability standing on ramps (*p* = 0.031) (Table 4). No other statistically significant differences were found.

## 4 Discussion

The purpose of this study was to investigate the effectiveness of enhancements to the Genium and Genium X3 MPK to improve safety during stumbling and everyday walking in a group of subjects with both unilateral and bilateral TFA, and specifically to improve the prosthetic experience of bilateral prosthesis users. The first hypothesis that the enhancements would reduce stumbles and falls and would improve gait stability and comfort in the sample was partially supported in that stumbles were greatly reduced, and comfort and stability of walking improved as measured by the SSQ and ADL-Q but not by PLUS-M, ABC, and *L*-test. The already low baseline number of falls was also reduced, but not to a level of statistical significance. The second hypothesis that the enhancements would improve patient-reported ease and safety of ADL completion in a group of bilateral users compared to a control group of unilateral users was also partially supported. The subjects with bilateral amputation showed significantly greater improvements in ADL-Q results compared to unilateral users, but not with the other outcome measures. Device performance did not diminish following implementation of the enhancements which is evident in the absence of significant aggregate or within-group declines in outcomes during the study period.

The most notable improvement overall was observed in stumble reduction. Stumbles significantly decreased 85% on aggregate. As fall frequency was quite low for this sample at baseline, the further decrease by 36% with the investigational Genium was not statistically significant. It is possible the reduction in stumbles may have translated to a statistically significant reduction in falls with a larger sample or longer study period because stumbles have led to falls in up to 57% of subjects in previous studies (27). The baseline frequency from this sample is slightly less than a group of (*n* = 19) subjects with unilateral TFA using C-Leg MPKs reported by Kahle et al. in 2008 who fell 1 ± 2 times in a similar period (28). Much attention has been given to fall prevention as a safety concern in recent years due to associated healthcare cost and mortality. The Centers for Disease Control and Prevention reports $50B in costs associated with non-fatal falls and $754M with fatal falls in the United States each year (29). A study by researchers from The Mayo Clinic found cost associated with falls by individuals with TFA to be more expensive than falls in the able-bodied, $25,652 compared to $18,091 respectively (30).

Pain has a significant influence on quality of life worldwide, but especially in the United States where it is the single most heavily-weighted dimension of the EQ-5D index (31). Chronic low back pain and residual limb pain are of unique interest for individuals with amputation, particularly those with TFA (16, 17). Mean differences reflected improvement for residual limb pain and low back pain on aggregate as a result of the enhancements. This was mainly driven by an improvement in unilateral subjects as shown by the mean differences in the age-matched unilateral group. The improvements approached the level of clinical meaningfulness for residual limb pain and reached a level of “much better” improvement for lower back pain (32). While not specifically addressed in the study, the pain reduction in unilateral subjects may have been the effect of improved everyday walking with increased symmetry, either in gait parameters or ground reaction forces, which in turn would have the potential to reduce low back pain and residual limb pain in unilateral subjects through more symmetric muscle activation. Improvements in gait symmetry is a common conclusion from studies with Genium use (4). The links between unilateral amputation, TFA, and increased rates of gait asymmetry, low back pain, and stump pain have been established previously (33). Changes to prosthetic knee joints are not expected to improve symmetries in bilateral subjects when both knee joints are the same. This finding likely explains why unilateral subjects reported improved comfort, exertion, and concentration while walking in the SSQ, while bilateral users did not. Pain is a subjective perceptive phenomenon involving cognitive processing; therefore, if an aspect of prosthetic gait is causing discomfort, then concentration is directed there (34). Pain has also negatively influenced perceived exertion levels in other reports (35, 36). Reductions in pain can have meaningful impact not only in the lives of individual patients but also the general healthcare system because pain is responsible for higher costs annually than diabetes and heart disease in the United States with the largest portion of that being attributable to low back pain (37). Similar trends are found throughout the world (38).

While improvements in several areas were noted for all subjects, the user experience of the bilateral group was particularly insightful. While improvements in ease and safety of ADL execution were noted in all categories of the comparative ADL-Q in the aggregate analysis, the bilateral group experienced significantly greater relative improvements in ease of performing ADLs in five categories and safety of performing ADLs in all seven categories than the age-matched unilateral users. This is similar to prior work by Kannenberg et al. where ease and safety of all ADL-Q categories improved or did not reduce in a sample of subjects with TFA comparing the C-Leg™ MPK to Genium, although that sample had only unilateral users (26). The ADL-Q serves as an informative tool regarding patient-reported ease and safety of ADL completion and research is needed to evaluate its psychometric properties. As with the ADL-Q ratings, the bilateral group also reported greater improvements in most items of the SSQ and particularly for walking safety and stability compared to the unilateral group. Although the changes were not statistically significant, it is important to note that at the end of the study these ratings were all very close to the maximum possible score with all subjects in the bilateral group reporting a 10 out of 10 for “overall prosthesis safety.” In contrast, the unilateral and bilateral groups were similar at baseline for “perceived exertion” and “concentration during walking,” but the mean rating of the unilateral group improved by over 45% for both items whereas the bilateral group mean remained constant.

The lack of significant differences between the bilateral and unilateral groups with the PLUS-M, ABC, and SSQ suggests similarity in patient-reported end-user experience in all areas tested except for actual physical performance measured with the *L*-test. The functional gap between individuals with history of bilateral and unilateral TFA was noted both at baseline and at the final assessment which is consistent with the literature (39, 11, 12). *L*-test times found here were similar to those reported by Deathe, et al. who observed (*n* = 46) subjects under the age of 55 with unilateral TTA and TFA ambulating with a prosthesis required an average of 25.4 ± 6.8 s to complete the *L*-Test (19). The Deathe sample included only unilateral subjects at both the TFA and TTA levels (19). A fall risk threshold of 25.5 s for healthy elderly subjects has been established, and there is no corresponding value for individuals with amputation (20). The bilateral group in this study performed near this threshold while the unilateral group performed well under (21). The observation of a persistent gap in physical performance between groups is probably due to the absence or presence of an unaffected leg, respectively, and supports the exemption of patients with bilateral limb loss from the MFCL K-level system (40). This exemption was also emphasized by the Lower Limb Prosthetics Inter-agency workgroup in 2017 (41).

The combination of improvements measured by the ADL-Q and SSQ and lack of any real decline in the validated patient-reported measures between-groups shows there were improvements to the patient experience of bilateral TFA users which are not or cannot be captured with currently available validated outcome measures. The validated measures used in this study, the *L*-test, PLUS-M, and ABC, while not an exhaustive list, evaluate diverse aspects of mobility, walking performance, and safety. This contrast of results between validated and study-specific OMs shows there is room for improvement in patient experience and product performance which can be achieved specifically when technological advancements are tailored to unique needs of sub-populations such as bilateral users, even when the users already appear to be functioning well. Further, current and commonly-used validated OMs may not be capturing discernable improvements in product function and aspects of daily life which are important to the end-user, specifically user-perceived safety, comfort, and ease of completing ADLs. Moreover, this discrepancy demonstrates the need for continual product improvement, even in the most advanced microprocessor-controlled components, to restore functional capacity and independence for patients—especially individuals with bilateral TFA.

### 4.1 Limitations

Since this study was supporting a product enhancement to determine its feasibility, sample size was based on stated needs of the product developers and not on a sample size calculation which is typically done in randomized clinical trials. Therefore, the overall sample of users with TFA (*n *= 25) and smaller sub-sample of bilateral subjects (*n *= 9) may have resulted in the study being underpowered. Further, users of the Genium and Genium X3 are usually MFCL K3 or higher and typically walk well, so there is not much room for improvement in functional performance which was evident in the baseline scores. While the mixed sample was necessary to determine the efficacy of this update, the heterogeneity somewhat limits the generalizability of aggregate findings for either group since they are clinically different.

A limitation of the Comparative ADL-Q is its direct comparison of recalled and concurrent experience which inherently introduces a bias. The experience with the existing prosthesis is recalled over an extended period whereas the experience with the experimental prosthesis is concurrent. Recalled ratings are often less accurate than concurrent ratings since the latter is fresh in the subjects' minds. This may also have affected the final question in the ABC regarding confidence walking on icy sidewalks because the baseline assessments occurred in spring and summer whereas final assessments occurred in winter. In this case the baseline assessment would be recalled and final assessment concurrent. This was the item of greatest improvement in the ABC, improving 46.9%–60.8%.

Subjects are also known to have an affinity for new technology which is referred to as pro-innovation bias (42). A common mitigation strategy is blinding. While blinding is typically not feasible in prosthetic studies due to the obvious differences in appearance of components, this enhancement was mostly internal and, hypothetically, could have been blinded (43). Lack of randomization or a crossover component also increases bias. However, the primary objective of the project was to confirm the feasibility of the enhancement. Therefore, the comparison of the enhancement between bilateral users and a control group of similar unilateral subjects was the most pragmatic solution.

An additional limitation was the use of the SSQ and ADL-Q in this study since the clinical meaning of unvalidated measures is obscure. Since this sample was high-functioning at baseline, a change in OMs validated in the population with limb loss was not expected and did not occur. However, the purpose of the enhancement was not necessarily to improve physical performance but rather to improve stability and comfort in several specific situations which would translate to improvements in perceived ease and safety of ADL completion. The ADL-Q has been used successfully in other studies comparing different MPKs (26). Further research to determine its psychometric properties may be warranted.

## 5 Conclusion

This study evaluated the implementation of a ruleset and hydraulics upgrade as well as bilateral parameter presets to the Genium™ and Genium-X3™. Marked reductions in stumbles, residual limb pain, and back pain were shown overall. These reductions were driven by the results of the subjects with unilateral amputation who also showed improvements in comfort, exertion, and concentration while walking. Improvements in patient-reported ease and safety of completing ADLs were shown overall and were driven by the results of the subjects with bilateral amputation who had significantly greater relative improvements compared to the unilateral users. Finally, performance of the MPKs did not decrease following the enhancement.

## Data Availability

The datasets presented in this article are not readily available because the dataset is property of Otto Bock Healthcare LP. Requests to access the datasets should be directed to Tyler Klenow, tyler.klenow@ottobock.com.
